# Complete genome sequence data of two *Salmonella enterica* subsp. *enterica* serovar Gallinarum: A 9R vaccine strain and a virulent Brazilian field strain

**DOI:** 10.1016/j.dib.2023.108959

**Published:** 2023-02-09

**Authors:** Ruy D. Chacón, Jorge L. Chacón, Manuel Ramírez, Carmen L. Rodríguez Cueva, Wilma Ursula Quispe-Rojas, César Bryan Reyes-Moreno, Claudete S. Astolfi-Ferreira, Antonio J. Piantino Ferreira

**Affiliations:** a Department of Pathology, School of Veterinary Medicine, University of São Paulo, Av. Prof. Orlando M. Paiva, 87, São Paulo 05508-270, Brazil; b Inter-Units Program in Biotechnology, University of São Paulo, Avenida Prof. Lineu Prestes, 2415, São Paulo 05508-900, Brazil; c Ceva Animal Health, R. Manoel Joaquim Filho, 303, São Paulo 13148-115, Brazil; d Área de Análisis Bioinformático, Centro de Investigaciones Tecnológicas, Biomédicas y Medioambientales, Jirón José Santos Chocano, 199, Bellavista 07006, Perú; e Laboratory of Biology and Molecular Genetics, Faculty of Veterinary Medicine, Universidad Nacional Mayor de San Marcos, Lima 15021, Peru; f Laboratory of Molecular Microbiology and Biotechnology, Faculty of Biological Sciences, Universidad Nacional Mayor de San Marcos, Lima 15081, Peru

**Keywords:** Fowl typhoid, Multilocus sequence typing, Serotype, Pathogenicity island, Virulence factor, Antimicrobial Resistance gene, Mobile genetic element, Prophages

## Abstract

*Salmonella* Gallinarum (SG) is a host-restricted enterobacteria and the causative agent of fowl typhoid in poultry. Here, we report the complete genomes of two strains belonging to this serotype. SA68 is a field strain isolated from the livers of dead hen carcasses of a commercial layer farm presenting high mortality located in São Paulo city, Brazil, in 1990. Strain 9R corresponds to a live attenuated SG commercial vaccine. DNA was extracted from pure cultures and subjected to whole genome sequencing (WGS) using the Ion Torrent PGM System. The assemblies reached lengths of 4,657,435 (SA68) and 4,657,471 (9R) base pairs. Complete genomes were deposited in GenBank under the accession numbers CP110192 (SA68) and CP110508 (9R). Both genomes were analyzed and compared in terms of molecular typing, antibiotic resistance genes, virulence genes, Salmonella pathogenic islands (SPIs), insertion sequences and prophages. The data obtained show many similarities in the genetic content, with the exception of the SPI-12 and CS54 pathogenic islands, which are exclusive to the field strain. The information generated will help to understand the virulence differences of field and vaccinal SG strains and can be used to perform evolutionary and epidemiologic studies.

**Specifications Table**

**Table:** 

Subject	Biological Sciences
Specific subject area	Bacteriology, Genomics
Type of data	Genome Sequence Data Table of general genomic characteristics Figures comparing the complete genome, pathogenicity islands and prophages Supplementary tables of antimicrobial resistance genes and virulence genes
How the data were acquired	Whole genome sequencing: Ion Torrent PGM System. Genome assembly: SeqMan NGen software (DNASTAR Lasergene). Circular genome visualization: BRIG v0.95 Genetic composition of SPIs and prophage visualization: Easyfig v2.2.2
Data format	Whole genome assembled and annotated Whole genome analysis
Description of data collection	The isolates were cultured on Xylose Lysine Tergitol 4 (XLT4) agar, and the Genomic DNA was isolated using the DNeasy Blood & Tissue Kit (Qiagen, Hilden, Germany). The raw reads were assembled using SeqMan NGen software (DNASTAR Lasergene, Madison, WI, USA) and annotated by NCBI Prokaryotic Genome Annotation Pipeline (PGAP v. 6.3).
Data source location	*Salmonella* Gallinarum SA68 strain was isolated in 1990 from liver fragments of dead chicken carcasses taken from layer hen farms in São Paulo, Brazil. *Salmonella* Gallinarum 9R strain was obtained from the Cevac® S. Gallinarum commercial vaccine. • Institution: University of São Paulo • City/Town/Region: São Paulo • Country: Brazil
Data accessibility	The assembled genome sequences were deposited in GenBank under the BioProject number PRJNA483415 and BioSample numbers: SAMN31392517 and SAMN31399569. Repository name: NCBI GenBank Accession Number Data identification numbers: CP110192 and CP110508 Direct link to the data: https://www.ncbi.nlm.nih.gov/nuccore/CP110192 https://www.ncbi.nlm.nih.gov/nuccore/CP110508

## Value of the Data

• The available complete genome sequencing data of field and vaccinal *Salmonella* Gallinarum strains provides insight into genetic differences among this avian serovar.
• The data also help to understand the increased virulence of field strains carrying additional virulence factors.
• The data can be used to perform comparative and evolutionary genomics for *Salmonella* Gallinarum and other *Salmonella* serovars.

## 1 Introduction

We aimed to sequence the complete genome of a field and a vaccinal strain of *Salmonella enterica* subsp. *enterica* serovar Gallinarum and compare their genomic characteristics.

## 2 Data Description

*Salmonella enterica* subsp. *enterica* serovar Gallinarum is a gram-negative *Enterobacteriaceae* that is flagellated and nonmotile. It is the major cause of fowl typhoid in mature birds, producing acute or chronic septicemic disease and significant economic losses in the poultry industry [1]. It is usually controlled by using live attenuated vaccines; however, it remains endemic to Asia and South America and causes outbreaks in developed countries [2]. Genetic and genomic approaches have shown differences in the virulence gene composition and the importance of horizontal gene transfer in genome evolution and host adaptation in chicken-associated serovars [3], [4], [5]. In this sense, the availability of SG genomes from distinct geographical and temporal locations allows for a deeper understanding of this pathogen [6,7].

The complete genomic data reported here include the whole-genome sequencing, assembly, annotation and comparative genomic data of two *Salmonella* isolates (SA68 and 9R) corresponding to field and vaccine strains. The raw reads from both genomes were trimmed and assembled. The genome assembly results, annotation, typification and genetic features and gene content are listed in Table 1.

**Table 1:** General genome characteristics of *Salmonella* Gallinarum SA68 and 9R strains.

Features	SA68	9R
NCBI BioSample no.	SAMN31392517	SAMN31399569
GenBank accession no.	CP110192	CP110508
No. of contigs	1	1
Total length (bp)	4,657,435	4,657,471
Total No. of CDS	4,412	4,448
TRNAs	75	73
NcRNAs	12	12
GC content (%)	52.21	52.12
Serogroup	D_1_	D_1_
MLST	78	331
CgMLST	37503	5460
CRISPR Arrays	1	1
Antibiotic Resistance genes	*aac(6′)-Iaa, acrAB, acrD, ampH, bacA, baeR, cpxA, crp, emrABR, golS, hns, kdpE, marA, mdsABC, mdtBC, mdtK, msbA, ompA, sdiA, tolC, yojI*	*aac(6′)-Iaa, acrAB, acrD, ampH, bacA, baeR, cpxA, crp, emrABR, golS, hns, kdpE, marA, mdsABC, mdtBC, mdtK, msbA, ompA, sdiA, tolC, yojI*
Virulence genes (N°)	132	131
SPI	SPI-1, 2, 3, 4, 5, 9, 10, 12, 13, 14, CS54, C63PI, PPIP *ssAD*	SPI-1, 2, 3, 4, 5, 9, 10, 13, 14, C63PI, PPIP *ssAD*
Insertion Sequences	ISKpn2, ISSty2, ISEcl10, MITEEc1	ISKpn2, ISSty2, ISEcl10, MITEEc1
Prophages	Complete: 1, ΦSA68 (∼Gifsy_2, NC_010393)	Complete: 1, ΦSA9R (∼Gifsy_2, NC_010393)

**Fig. 1:**
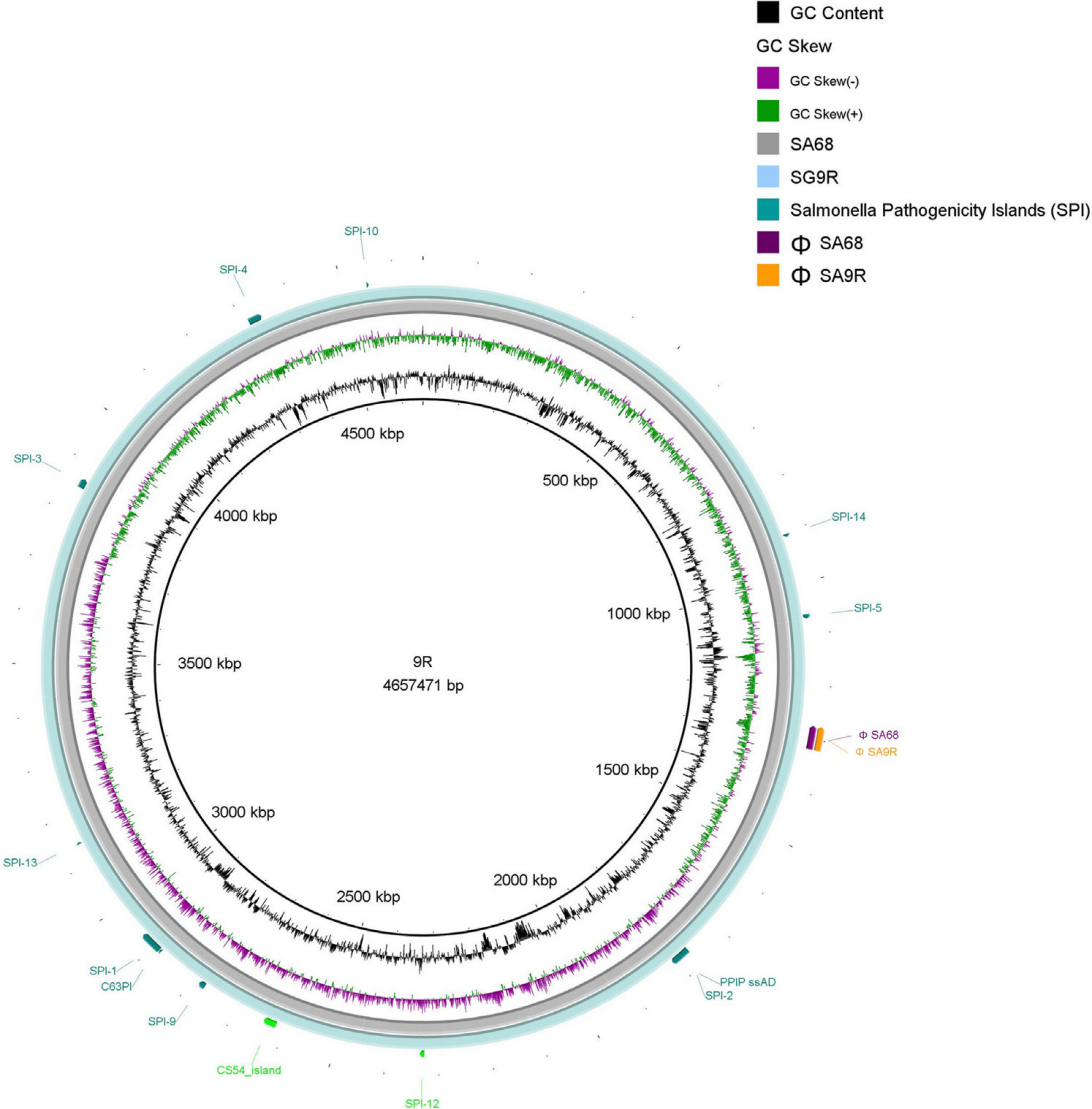
Genome comparison between *Salmonella* Gallinarum strains SA68 (gray ring) and 9R (sky blue ring). The *Salmonella* pathogenicity islands and prophages were mapped and identified as colored bars and lines on the outer side of the rings. This figure was generated using BRIG v0.95. [14].

**Fig. 2:**
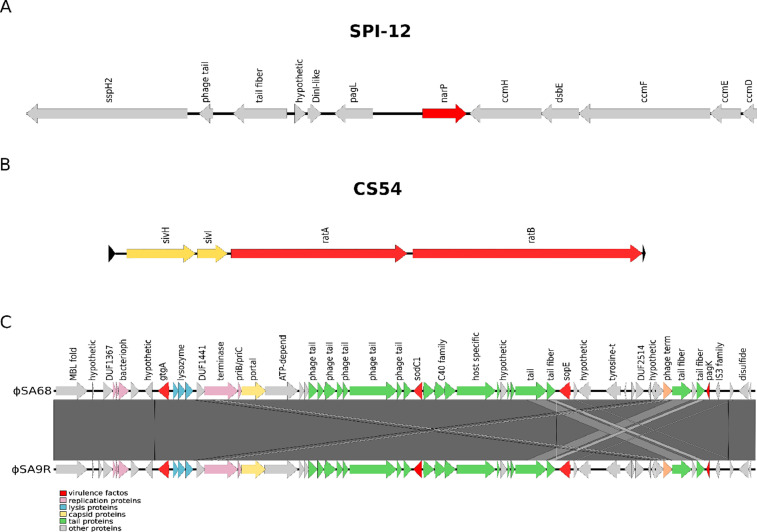
A) Genetic organization of the virulence factors in *Salmonella* pathogenicity island 12 (SPI-12). B) Genetic organization of the virulence factors in *Salmonella* pathogenicity island CS54 (CS54). C) Genetic organization of the virulence factors in prophages ΦSA68 and ΦSA9R. Virulence factor genes are colored with red filled arrows. This figure was generated using Easyfig v2.2.2 [15].

The comparative genomic analysis identified typification differences between MLSTs (ST78, ST331) and cgMLST (37503, 5460). Both isolates shared thirteen *Salmonella* pathogenic islands (SPIs). However, SA68 presented 2 additional SPIs (Fig. 1, Fig. 2A, Fig. 2B), with associated functions of nitrate/nitrite response regulation and macrophage survival (SPI-12, of 9,343 bp length) [8] carrying the gene *narP* (Fig. 2A) and intestinal colonization and persistence determinants (CS54, of 25,184 bp length) [9] carrying the genes *ratA, ratB, sivH*, and *sivI* (Fig. 1, Fig. 2B). Both genomes presented 27 antimicrobial resistance genes (Table 1; Table S1) [10] and 132 and 131 virulence genes were identified in SA68 and 9R, respectively (Table 1; Table S2) [11]. We also detected mobile genetic elements with common insertion sequences (ISKpn2, ISSty2, ISEcl10, and MITEEc1) and one complete prophage each (ΦSA68 and ΦSA9R) (Fig. 1, Fig. 2C), which presented a similar genetic content to the reported ΦGifsy-2 [12], a prophage known to have a role in infection [13], carrying the virulence factors *gtgA, sodC1, sopE*, and *pagK* in ΦSA68 and ΦSA9R (Fig. 2C).

## 3 Experimental Design, Materials and Methods

### 3.1 Bacterial isolation, genomic DNA extraction and sequencing

Field strain SA68 originated from a liver sample and was cultured in tetrathionate for 48 hrs at 37°C and then cultured in xylose lysine tergitol 4 (XLT4) agar for 24 h at 37°C. Typical colonies were detected and subjected to biochemical testing using Enterokit B (Probac, SP, Brazil). Vaccinal strain SG 9R was obtained from Cevac® S. Gallinarum lyophilized live vaccine. Colonies were cultured in LB broth for 18 hrs at 37°C for DNA extraction using the DNeasy Blood & Tissue Kit (Qiagen, Hilden, Germany). Samples were submitted for whole genome sequencing. Libraries were prepared using the Ion Torrent RNA-Seq kit and sequenced on the Ion Torrent PGM.

### 3.2 Genome assembly, annotation, descriptive and comparative genomic analysis

The nucleic acid sequences of each sample were assembled using SeqMan NGen software (DNASTAR Lasergene, Madison, WI, USA). The S. Gallinarum 287/91 strain (GenBank Accession Number: AM933173) was used as the reference scaffold for the templated assembly. The Q-score for all sequences included in the assembly was Q>28. The average read length was ∼140 bp. The genomes were annotated by NCBI Prokaryotic Genome Annotation Pipeline (PGAP v. 6.3) [16].

Molecular typing for multilocus sequence type (MLST) and cgMLST were detected by mlst 2.11 (https://github.com/tseemann/mlst). *In silico* serotyping was performed with SeqSero2 v1.1.0. [17]. We screened for the presence of horizontally acquired antibiotic resistance genes and virulence factors by using ABRicate v1.0.1 (https://github.com/tseemann/abricate) according to the Comprehensive Antibiotic Resistance Database (CARD) [10] and the Virulence Factor Database (VFDB) [11]. *Salmonella* pathogenic islands were identified with SPIFinder v2.0 by 60% of minimum coverage [18]. Detection of insertion sequences was performed with Mobile Element Finder v1.0.3 [19]. The detection of prophages was performed with the Phaster search tool [20]. Both genomes were mapped against the reference genome NCTC8325 (NC_007795.1) and the genetic organization of SPIs and prophages was illustrated using BRIG v0.95 [14] and Easyfig [15].

## Ethics Statements

The study was conducted according to the guidelines of the Declaration of Helsinki and approved by the Ethics Commission on Animal Use of the School of Veterinary Medicine, University of São Paulo (FMVZUSP), under CEUAVET protocol no. 5082200218 on 18 November 2021.

## CRediT Author Statement

**Ruy D. Chacón:** Conceptualization, Methodology, Software, Writing – Original draft preparation; **Jorge L. Chacón:** Conceptualization, Methodology, Supervision; **Manuel Ramírez:** Methodology, Software, Writing – Original draft preparation; **Carmen L. Rodríguez Cueva:** Methodology, Software, Writing – Original draft preparation; **Wilma Ursula Quispe-Rojas:** Methodology, Software, Writing – Original draft preparation; **César B. Reyes-Moreno:** Methodology, Software, Writing – Original draft preparation; **Claudete S. Astolfi-Ferreira:** Methodology, Supervision; **Antonio J. Piantino Ferreira:** Conceptualization, Methodology, Supervision.

## Declaration of Competing Interest

The authors declare that they have no known competing financial interests or personal relationships that could have appeared to influence the work reported in this paper.

## Data Availability

Salmonella enterica subsp. enterica Genome sequencing and assembly (Original data) (BioProject NCBI).

## Appendix Supplementary materials

Image, application 1

Image, application 2

Image, application 3

Image, application 4
